# Familial Papillary Thyroid Carcinoma: A Retrospective Analysis

**DOI:** 10.1155/2011/948786

**Published:** 2011-10-25

**Authors:** Thomas J. McDonald, Albert A. Driedger, Bertha M. Garcia, Stanislaus H. M. Van Uum, Irina Rachinsky, Vijaya Chevendra, Daniel Breadner, Richard Feinn, Stephen J. Walsh, Carl D. Malchoff

**Affiliations:** ^1^Department of Medicine, University of Western Ontario London, ON, Canada N6A 5A5; ^2^Department of Nuclear Medicine, University of Western Ontario London, ON, Canada N6A 4S2; ^3^Department of Pathology, University of Western Ontario London, ON, Canada N6A 5A5; ^4^Department of Medicine, University of Connecticut Health Centre, Farmington, CT 06107, USA

## Abstract

*Background*. Whether or not the familial form of papillary thyroid carcinoma is more aggressive than the sporadic form of the disease remains controversial. *Methods*. To explore this question and whether or not increased aggressiveness is more apparent in families with multiple affected members, we performed a chi square by trend analysis on our patients clinical and pathologic data comparing: first degree families with three or more affected members versus first degree families with two affected members versus sporadic cases of papillary thyroid carcinoma. *Results*. No statistically significant trends were seen for any presenting surgical pathology parameter, age at presentation, length of follow-up or gender distribution. The familial groups exhibited significant trends for higher rates of reoperation (*P* = 0.05) and/or requiring additional radioactive iodine therapy (*P* = 0.03), distant metastases (*P* = 0.003) and deaths (*P* = 0.01). These aggressive features were most apparent in certain families with three or more affected members. *Conclusions*. Using the chi square by trend analysis, a significant trend was seen for the familial form of papillary thyroid cancer to possess more aggressive features than the sporadic disease. Prompt recognition of the familial nature of the disease may provide earlier diagnosis and treatment in similarly affected family members.

## 1. Introduction

Early reports of well-differentiated nonmedullary thyroid carcinoma clustering in families [1–7], and studies utilizing population and hospital data bases [8–13] demonstrating that such cases occurred in families more often than could be explained by chance have led to the general acceptance of the existence of a familial form of the disease. Most reported familial cases are papillary carcinoma, including familial papillary thyroid microcarcinoma [14]. A hereditary basis for this familial nonmedullary thyroid cancer is postulated, but no specific genetic defect has been established as yet [15–17], and the suggestion has been made that the disease may result from a heterogeneous form of inheritance or the interaction of susceptibility genes with unidentified environmental factors [17–19].

Aspects of the natural history of familial papillary thyroid carcinoma (FPTC) remain unclear, and specifically controversial is whether or not the familial form of the disease is more aggressive than the sporadic form. In certain reports, the familial form of the disease was associated with an earlier age at diagnosis [3, 7], a higher than usual ratio of male to female cases [14, 20], a greater incidence of spread outside the thyroid gland, [3, 14, 20, 21] and a greater rate of persistence/recurrence [7, 14, 20–23]. However, a number of other studies did not confirm the presence of these measures of aggressiveness, including a recent retrospective study [24], and, in a review of published studies, Loh [25] did not find conclusive evidence for increased aggressiveness in the familial form of the disease. Charkes [26] has pointed out that most reports consist predominantly of families with only two affected members, and in his analysis a high percentage (62–69%) of patients in two member families may suffer from the sporadic rather than the familial form of the disease. Hence, a number of reports may have included the data from a significant number of sporadic cases in their analysis of the characteristics of familial cases. In contrast, kindred's with three or more affected members overwhelmingly (96%) represent the familial form of the disease [26], leading Charkes to suggest that these kindred's be used for clinical and genetic investigations. Further, as FPTC may be a heterogeneous entity, there may be significant differences in the degree of aggressiveness between different families within any given report of cases, rendering comparisons in series with small numbers of families difficult.

In this communication, we describe a retrospective analysis of our experience with sporadic versus FPTC cases seen at the University of Western Ontario. Given Charkes' analysis and recommendations [26], we chose a statistical approach designed to determine the presence or absence of a trend for the occurrence of more aggressive disease in families with three or more affected members by comparing the clinical and pathology findings of sporadic cases versus first degree families with two affected members versus first degree families with three or more affected members. This approach was chosen to address the issue of mistaking sporadic cases for familial cases in two member families, and to address the possibility that abnormal genes causing a higher prevalence of disease may also result in more aggressive disease. We further explored the possible presence of disease heterogeneity occurring between FPTC families (i.e., the possible presence of an aggressive genotype clustering in certain families while other families contain members with a genotype resulting in more indolent disease).

## 2. Materials and Methods

Patients attending our clinics at the University of Western Ontario with an established diagnosis of nonmedullary thyroid cancer were questioned regarding the presence or absence of similarly affected family members. We attempted to confirm the diagnosis of identified additional affected members by obtaining their medical and pathology records and when possible reviewed these family members in our clinic. The surgical pathology, therapeutic, and followup data on all cases were entered into an access database organized into four categories: (1) sporadic cases, (2) familial cases with affected second degree or higher relatives, (3) familial cases with first degree relatives in families containing only two affected members, and (4) familial cases with first degree relatives in families containing three or more similarly affected individuals. For all cases, we entered into the data base details of the clinical course (including evidence of recurrence/persistence of disease such as reoperation, requirement for additional radioactive iodine therapy, etc.) and surgical pathology results including histological tumour type; tumour number, size, and site; the presence or absence of (a) tumour penetration of thyroid capsule and/or presence in perithyroidal tissue, (b) vascular or lymphatic invasion, (c) diffuse lymphocytic infiltration, (d) multifocal disease, (e) bilateral disease, (f) cervical nodal metastases, and (g) distant metastases. The presence of distant metastatic disease was established by surgical removal or biopsy of thyroid cancerous tissue or by persistent radio-iodine uptake in abnormal sites (e.g., lung, bone, CNS, etc.) with no other known cause. Reoperation was defined as any surgery carried out to remove proven recurrent/persistent thyroid cancer after the initial surgical therapy of a total thyroidectomy (one or two stage procedure). Deaths were attributed to thyroid cancer only if other causes of death could be ruled out. Radioactive iodine therapy was administered in accordance with the American Thyroid Association guidelines. 

Within the ascertainment time of case acquisition, the data base contained 698 confirmed cases of nonmedullary well-differentiated thyroid cancer. Of these cases, 664 were papillary thyroid cancer (Table 1), and all cases unequivocally established as occurring in a familial setting were classified pathologically as papillary thyroid cancer. Hence the term familial papillary thyroid cancer (FPTC) is used in this paper. None of the established FPTC families had any evidence of additional tumours or clinical features to suggest the presence of a familial syndrome [18, 27, 28] characterized by a predominance of nonthyroidal tumours, for example, familial adenomatous polyposis, PTEN-hamartoma syndrome, Carney complex, and so forth. This study was approved by the Ethics Review Board for Human Research at the University of Western Ontario.

 Sixty patients presenting in our clinic gave a history of first degree relatives being similarly affected with PTC; by history, 22 families contained 3 or more similarly affected first degree relatives with PTC (data was adequate for analysis on 52 patients), and 38 families had only 2 affected members (data was adequate for analysis on 55 of these patients). An additional 33 families with similarly affected second degree or higher relatives were identified (data was adequate for analysis on 36 of these patients). These patients form a data set derived from families classified by historical evidence alone without the necessity of having obtained confirmatory pathology on all family members. This data set (the history-defined data set) was analyzed as described below, comparing four groups: first degree relative families with three or more affected members, versus first degree relative families with two affected members, versus second degree or greater relative families with affected members, versus 521 concurrent sporadic cases. This history-defined data set was analyzed as described below, and the results are presented in Table 5.

 On review of the pathology data obtained on additional identified family members, we were able to confirm (in all members) a pathology proven status of 14 first degree relative families with 3 or more affected members (41 members of this group had adequate data for analysis) and 30 first degree families with only 2 affected members (50 members of this group had adequate data for analysis). We performed an identical statistical analysis on this data set (the pathology-defined data set) comparing the presenting and clinical followup data on three groups: first degree families containing 3 or more affected members versus first degree family members containing 2 affected members versus 521 PTC patients with sporadic disease seen concurrently. The results of this analysis on the pathology-defined data set are presented in detail in the text and in Tables 2–4.

 To assess the validity of classifying families based on historical evidence alone without requiring pathological confirmation of the presence of PTC in all additional identified members, we subjected the data sets obtained from both the history-defined families and the pathology-defined families to identical statistical analyses. The results from the analyses of the two data sets yielded identical statistical results. For the sake of brevity, only the results of the pathology-defined data set are given in detail in the text and in Tables 2, 3, and 4. Table 5 displays the most relevant data from the history-defined data set together with a comparison of the statistical results derived from the analysis of both data sets. 

For statistical analysis, mean ages were contrasted using analysis of variance, and distribution of gender in the groups was compared using the chi-square test for contingency tables. The method of statistical analysis was chi square by trend for the frequency of the presenting surgical pathology parameters, metastases, and for the followup data including subsequent required treatments relative to the extent of familial incidence of papillary thyroid cancer; the Cochran-Armitage test was used for evaluation. Due to small counts for some events, the “exact” version of the Cochran-Armitage test was utilized. To examine for heterogeneity of disease between individual families in the first degree relative categories, the log linear model with Poisson count was used to model disease deaths and distant metastases by family memberships. *P* values for the exact test were calculated using StatXact software (Cytel Software Corporation, Cambridge, Mass., USA). Statistical significance was accepted for *P*-values ≤0.05.

## 3. Results

Tables 1(a) and 1(b) display the diagnostic details for all 698 patients with nonmedullary thyroid cancer in the database. The overwhelming majority of cases, 664 patients (95.1%), were diagnosed with papillary carcinoma, 13 cases contained areas of the tall cell variant, 15 cases contained areas of the Oxyphil cell variant of PTC, and 10 had areas of solid type growth patterns. The non-PTC cases were classified as 19 follicular carcinomas, 14 Hurthle cell carcinomas (11 of which were classified as minimally invasive), and one clear cell carcinoma. There were four sets of apparently identical twins. Three of the four sets had concordant diagnoses of PTC (all made within one year of each other); the fourth set has one twin with proven PTC, the other twin is currently under ongoing investigation for nodular disease, and a further sibling has treated PTC. Three of the four sets of twins occur in families with additional members with PTC, the fourth set occurs in a family with prominent nodular disease. Ten of the 13 cases with foci of the tall cell variant PTC occurred in the nonfamilial cases, one in a first degree relative (3 or greater) family member who did not demonstrate any aggressive features at surgery nor on followup, and in two members of the second degree or greater relative families (both of which had distant metastases). There were 10 cases of PTC with foci of solid type growth pattern, all of which occurred in sporadic PTC cases. None of the familial cases had prior head and neck radiation. 

The demographics of the nonfamilial and the PTC familial cases are shown in Table 2. There was no statistically significant difference in the age at diagnosis between the nonfamilial and familial cases, and a predominance of females to males was seen in all groups with no statistically significant differences in sex distribution between the groups. Similarly, no significant difference between the familial and nonfamilial cases was found in the percentage of patients aged 45 or older at diagnosis (Table 2), nor was there any significant difference in the average length of followup. 

 There were no statistically significant differences seen for any of the surgical pathology parameters analyzed (Table 3) including tumour size greater than 3 cm, presence of tumour in perithyroidal tissue/penetration of thyroid capsule, multifocal or bilateral disease, vascular or lymphatic invasion, and lymphocytic infiltration. Similarly, there was no statistically significant difference in the presence of cervical nodal metastases between the groups (Table 4). In contrast, the presence of distant metastases was statistically significantly higher in the familial cases than in the sporadic cases (*P* = 0.003), the distant metastases being seen particularly in families with three or more affected members (Table 4). The familial group had statistically significant increased rates of recurrent/persistent disease as judged by the requirement for reoperation (*P* = 0.05), the requirement of additional radio-active iodine therapy administered at least two years after the initial therapy (*P* = 0.03), or a combination of these two therapeutic modalities (*P* = 0.03). Although the number of deaths due to thyroid cancer was small over the time period of case ascertainment, with the statistical approach used there was a statistically significant trend for greater mortality (*P* = 0.01) in the familial groups. The two deaths in the first degree relative families with 3 or more affected members group occurred in one family which had multiple members affected by the disease (a third member is thought to have died of the disease but the cause of death could not be established with certainty); both patients presented late in the course of the disease (as was the case for the deaths occurring in both the familial and sporadic groups). There were 24 cases with distant metastases (Table 4), 16 in the sporadic disease group and 8 in the familial group. The presence of distant disease was discovered close to or at the time of diagnosis of thyroid cancer in almost all of the patients (a) in the sporadic disease group, 12 cases had the distant spread discovered at the time of diagnosis of thyroid cancer, 3 cases within the first year, and in one case the precise time elapsed was uncertain, (b) in the 6 familial cases found in the three or more member families group, 3 had the distant spread discovered at the time of diagnosis of thyroid cancer, 2 patients within one year, and within two years in 1 patient, and (c) in the families with two affected members only, one patient was diagnosed with distant metastases within one year and one case eighteen years after the original diagnosis of thyroid cancer. An additional analysis, performed to explore for heterogeneity in our first degree families, did not find any statistical evidence for the presence of heterogeneity for either deaths (*P* = 0.25) or distant metastases (*P* = 0.54). However, it is noted that the number of events analyzed was small; perhaps a greater number of events would be necessary to perform an adequate analysis.

Table 5 presents the most relevant data from the history-defined family groups data set and compares the statistical results obtained in this group to the results obtained on the data set of the pathology-defined family groups. In agreement with the analysis of the pathology-defined family data set, there were no statistically significant trend differences seen in any of the demographic data (age at diagnosis, sex distribution, mean followup time, and percentage of patients diagnosed after the age of 45 years). There were no significant differences seen in any of the surgical pathology data although the history-defined data set analysis results approached significance in certain parameters: tumour in perithyroidal tissue/thyroid capsule penetration (*P* = 0.08), multifocal disease (*P* = 0.06), and bilateral disease (0.07). Statistically significant trends also were seen for distant metastases (0.002), deaths (0.02), reoperation (0.03), and requirement for additional RAI (*P* = 0.01). Hence, the statistical results are consistent using either approach to defining the family groups.

## 4. Discussion

Despite the presence of a number of reports on the subject, it remains controversial as to whether the familial form of PTC is more aggressive than the sporadic form of the disease. Possible reasons for this uncertainly include the potential for PTC to reappear long after the initial therapy, the relatively infrequent occurrence of the familial form of the disease (most studies report an incidence of approximately 5% of PTC cases, but one recent prospective study (32) reports an incidence of 9.4% of PTC being familial), the unrecognized inclusion of sporadic cases in the analysis of familial disease characteristics and the possibility of FPTC being a heterogeneous disease resulting in varying degrees of aggressiveness occurring between different families. In this study, we used a different statistical approach, the Chi square for trend analysis, comparing the characteristics of sporadic cases versus cases in first degree relative families with only 2 affected members versus cases in first degree families with 3 or more affected members. With this method of data analysis, our study found a statistically significant trend for the familial disease to exhibit more aggressiveness in certain disease characteristics and to require further intervention after the initial therapy. Most studies have defined their family groups using historical data derived from presenting patients. We compared the data derived from using a historical approach (history defined) to defining the family groups (i.e., the number of affected patients in a family) to that derived from family groups defined by requiring pathological confirmation of all included members (pathology defined) by subjecting both data sets to identical analyses. This comparison yielded identical statistical results; it appears that with an important disease entity such as PTC, families possess a good understanding of the family medical history. 

The data for this study is derived from patients residing in an ethnically diverse population in which, similar to other regions, the incidence of PTC is clearly rising [29]. PTC accounted for 95.1% of our cases of well-differentiated thyroid cancer and the female-to-male ratio of cases is similar to that reported in other areas. We did not see any significant difference in the proportion of female-to-male cases between the sporadic and familial cases in agreement with some reports [7, 24, 30] but in contrast to others [14, 20] which found a higher than expected proportion of males in the familial cases. Similar to other reports [6, 20], we did not see a significant trend towards an earlier age at diagnosis of FPTC, but other studies do report an earlier age at diagnosis of the familial disease [3, 7, 30]. We did not find statistically significant differences between the nonfamilial and familial cases in any of the surgical pathology parameters. It had been reported that the familial form of the disease has a greater incidence of cervical nodal metastases [3, 20–22], but we did not find any such increase. The multifocal nature of the disease was a very common finding in all of our cases (present in 52.2 to 65.5%), as was the presence of bilateral disease in all groups, but there were no significant differences seen between the familial and sporadic cases; a higher incidence of multifocality has been reported in certain series [6, 7, 14, 20, 27]. We did not see a trend to a larger size of the primary tumour in the familial cases nor, somewhat surprisingly, any difference in the finding of vascular or lymphatic invasion by tumour. Although there was a greater incidence of tumour presence in perithyroidal tissues in the three or more member families, this was not statistically significantly different. 

On analysis of the data on the clinical findings and course seen at or within a few years of the initial diagnosis (Table 4), we found a statistically significant trend for the familial form of the disease to exhibit certain aggressive features. A greater incidence of distant metastases (*P* = 0.03) was seen in the familial form of the disease, being particularly prominent in the families with three or more affected members, the diagnosis being made in all of these cases within 2 years of the initial diagnosis of thyroid cancer. After the initial diagnosis and treatment, the familial cases also had a significantly greater requirement for reoperation (*P* = 0.05) and, beyond 2 years of followup from the initial therapy, had a significantly greater need for further treatment with radio-active iodine (*P* = 0.03). Of note, as our mean followup times are short (ranging from 4.22 to 5.37 years) in all groups, these findings probably represent persistence of disease rather than recurrence. These measures of aggressiveness cannot be readily explained by a greater incidence of tumours with histological features associated with a more aggressive clinical course. There was only one first degree relative family member with the tall cell variant of the disease, and this patient did not display any aggressive features at surgery nor on clinical followup to date. Similarly, the presence of foci of the solid-type growth pattern was seen only in the nonfamilial cases. Nor can the trend for these measures of aggressiveness in the familial form of the disease be explained by an increased age at diagnosis since this factor was not statistically different between groups. 

 Triponez et al. [31] have reported that the survival rate of affected members of families with three or more members was significantly shorter than those in families with only two affected members; this would be consistent with more aggressive disease being present in families with three or more affected members. In agreement with Charkes analysis [26], features of aggressive disease in this study were most frequently seen in the first degree relative families with three or more affected members, and these appeared to cluster in certain families; the distant metastases were present in only 5 of the 14 families, and the two deaths occurred in one family. An analysis to explore for the possible presence of heterogeneity in our families did not produce any statistical evidence for such heterogeneity. However, the number of events in our data may not have been sufficient to yield an accurate appraisal of this possibility. With regard to the issue of heterogeneity, Moses et al. [30] have recently published a prospective study which found a similar heterogeneity of numbers and types of somatic mutations in both the sporadic and familial forms of well-differentiated thyroid cancer cases; no evidence was seen for a clear genotype-phenotype distribution based on hereditary disposition. This question remains open for further genetic investigation.

 The cases with features of aggressive disease were most often present in the first member diagnosed in the family. Once the disease was recognized in the family, other affected members were usually diagnosed at an earlier stage of the process and have fared better on followup to date. For example, in one of our families, the initial case was diagnosed late in the disease with multiple distant metastases as well as inoperable local cervical disease. Two offspring in this family were screened for disease and both found to have micro-papillary carcinomas which had penetrated the thyroid capsule and spread to cervical nodes. Following surgery and radio-iodine therapy, both are currently considered disease-free (negative stimulated thyroglobulin levels). Similarly, in an earlier report by Triponez et al. [31], it was found that survival times were significantly shorter for affected members treated before the familial nature of the disease was recognized. Further, Moses et al. [30] found a statistically significant earlier age at diagnosis in familial patients and attributes this to other family members being made aware of the familial nature of the disease and, hence, presenting at an earlier stage in the disease process. We did not see a significantly earlier age at diagnosis, but, by focusing attention on the family history and encouraging family members to undergo screening for the disease, we well may have altered the outcomes in these patients for the better. A potential problem biasing results in the opposite direction would result from familial patients being more motivated to present for followup procedures than patients with sporadic PTC. If this occurred, less disease may have been discovered in our sporadic PTC patients. 

This study is retrospective in nature, which would be expected to limit the completeness and accuracy of data collection in patients treated in the earlier periods of this study. Further, there have been significant technological advances over the period of data collection which has altered our ability to detect the presence of persistent/recurrent disease (e.g., advances in imaging, greater sensitivity and specificity of thyroglobulin assays, and the determination of stimulated thyroglobulin levels), rendering comparisons over time somewhat more difficult. Although the majority of our cases were seen in the recent past, a minority of our cases were first diagnosed and treated at a time predating current technical abilities. Our mean followup times are too short to allow the comparison of clinical outcomes beyond the period of a few years following the initial diagnosis. The detection of persistent/recurrence of PTC may occur many years after the initial diagnosis and therapy; microscopic disease below the limits of detection at the time of initial therapy may become apparent only with the passage of an appreciable period of time. It is possible that differences in longer-term clinical outcomes between sporadic and familial disease may differ from those seen with the shorter time of followup in this study. However, the logistics of carrying out a long-term prospective clinical research project to explore this question are quite daunting. It is probable that ongoing research will yield a better understanding of the disease with the use of improved genetic diagnostic methods and imaging techniques. A more precise approach to early diagnosis, treatment and followup, may render such long-term clinical studies unnecessary. 

In summary, using a statistical approach (Chi square by trend) that compared PTC in sporadic cases versus first degree relative families with two affected members versus first degree relative families with three or more affected members, this study found evidence that the familial form of the disease exhibits significantly more aggressive features (a) the presence of distant metastases found at or shortly following the diagnosis of thyroid cancer and (b) the requirement for reoperation or further treatment with radio-active iodine following the initial treatment period. As these features were predominantly seen in the first degree families with three or more affected members, our results support the findings and the suggestion of Charkes [26] that genetic and clinical investigations should focus on families with multiple affected members. In agreement with Triponez et al. [31], patients, diagnosed subsequent to the recognition of the familial nature of the disease, tend to have better outcomes. This underlines the importance of early diagnosis and treatment for optimal outcomes. Until reliable genetic testing becomes available, obtaining a good family history is of great importance in the recognition of the familial nature of well differentiated thyroid cancer cases. Although the use of ultrasound as a routine screening device is controversial, it is well recognized that significantly more nodules are seen on ultrasound examination than are found on clinical examination, and that nodule size as a predictor of the presence or absence of malignancy is unreliable. In cases where the presence of FPTC is suspected, the use of ultrasound for screening and the use of FNA under ultrasound guidance on small lesions may result in earlier diagnosis and better outcomes. Although this may be a somewhat more aggressive diagnostic approach, it should be considered when investigating potential or known familial cases.

##  Conflict of Interests

The authors have no competing financial interests to disclose.

## Figures and Tables

**Table:** (a)

Type	Number of cases	**%**
Follicular carcinoma	19	2.7
Hurthle cell carcinoma	14	2.0
Clear cell carcinoma	1	0.1
Papillary thyroid carcinoma	664	95.1

Total	698	

**Table:** (b)

Subtype	Number of cases
Classical/follicular	626
Foci of oxyphil cell variant	15
Foci of tall cell variant	13
Foci of solid cell growth pattern	10

Total	664

**Table 2:** Demographics of nonfamilial and familial cases.

	Nonfamilial	1st Degree, 2 members	1st Degree, 3 or more members	*P *value
No. of patients	521	50	41	
Mean age at diagnosis (yrs ± SEM)	44.3 ± 0.65	40.12 ± 1.9	43.2 ± 1.9	0.14
No. of patients with diagnosis at age >45	223 (42.8%)	17 (34.0%)	17 (41.5%)	0.55
No. of females	414 (79.5%)	42 (84.0%)	30 (73.2%)	0.65
Mean length of followup (yrs ± SEM)	4.22 ± 0.18	4.83 ± 0.69	5.37 ± 0.83	0.18

**Table 3:** Surgical pathology results of nonfamilial and familial cases.

	Nonfamilial	1st Degree,2 members	1st Degree, 3 or more members	*P *value
No. of patients	521	50	41	
Tumour > 3.0 cm	126 (24.2%)	12 (24.0%)	8 (19.5%)	0.61
Tumour in perithyroidal tissue/thyroid capsule penetration	87 (16.7%)	8 (16.0%)	11 (26.8%)	0.17
Multifocal disease	272 (52.2%)	31 (62.0%)	23 (56.1%)	0.34
Bilateral disease	170 (32.6%)	20 (40.0%)	16 (39.0)	0.25
Vascular/lymphatic invasion	58 (11.1%)	7 (14.0)	5 (12.2%)	0.73
Lymphocytic infiltration	215 (41.3%)	19 (38.0%)	16 (39.0%)	0.71

**Table 4:** Metastases and clinical course.

	Nonfamilial	1st Degree, 2 members	1st Degree, 3 or more members	*P* value
No. of patients	521	50	41	
Cervical nodes positive	98 (18.8%)	12 (24.0%)	10 (24.4%)	0.27
Distant metastases	16 (3.1%)	2 (4.0%)	6 (14.6%)	0.003
Additional surgery	27 (5.2%)	2 (4.0%)	6 (14.6%)	0.05
Additional RAI >2 yrs after initial treatment	13 (2.5%)	4 (8.0%)	3 (7.3%)	0.03
Reoperation or additional RAI, 2 yrs after initial treatment	34 (6.5%)	6 (12.0%)	6 (14.6%)	0.03
Deaths due to disease	2 (0.4%)	1 (2.0%)	2 (4.9%)	0.01

**Table 5:** Selected data on history-defined families. Comparison of statistical analysis on history-defined and pathology-defined families.

	Nonfamilial	2nd degree or higher members	1st Degree, 2 members	1st Degree, 3 or more members	*P*-value history group	*P*-value pathology proven group
No. of patients	521	36	55	52		
Tumour in perithyroidal tissue/thyroid Capsule penetration	87 (16.7%)	8 (22.2%)	9 (16.4%)	15 (28.8%)	0.08	0.17
Multifocal	272 (52.2%)	22 (61.1%)	36 (65.5%)	31 (59.6%)	0.06	0.34
Vascular/lymphatic invasion	58 (11.1%)	6 (16.7%)	8 (14.5%)	6 (11.5%)	0.56	0.73
Cervical nodes positive	98 (18.8%)	8 (22.2%)	15 (27.3%)	13 (25.0%)	0.10	0.27
Distant metastases	16 (3.1%)	2 (5.6%)	3 (5.5%)	7 (13.5%)	0.002	0.003
Deaths	2 (0.4%)	1 (2.1%)	1 (1.8%)	2 (3.1%)	0.02	0.01
Reoperation	27 (5.2%)	2 (5.6%)	2 (3.6%)	8 (15.4%)	0.03	0.05
RAI, Additional treatment, 2 years after initial treatment	13 (2.5%)	2 (5.6%)	4 (7.3%)	4 (7.7%)	0.01	0.03
